# Flexible Ti_3_C_2_T_*x*_/(Aramid Nanofiber/PVA) Composite Films for Superior Electromagnetic Interference Shielding

**DOI:** 10.34133/2022/9780290

**Published:** 2022-02-02

**Authors:** Yali Zhang, Zhonglei Ma, Kunpeng Ruan, Junwei Gu

**Affiliations:** Shaanxi Key Laboratory of Macromolecular Science and Technology, School of Chemistry and Chemical Engineering, Northwestern Polytechnical University, Xi'an, Shaanxi 710072, China

## Abstract

Multifunctional electromagnetic interference (EMI) shielding materials would solve electromagnetic radiation and pollution problems from electronic devices. Herein, the directional freeze-drying technology is utilized to prepare the aramid nanofiber/polyvinyl alcohol aerogel with a directionally porous structure (D-ANF/PVA), and the Ti_3_C_2_T_*x*_ dispersion is fully immersed into the D-ANF/PVA aerogel *via* ultrasonication and vacuum-assisted impregnation. Ti_3_C_2_T_*x*_/(ANF/PVA) EMI shielding composite films with directionally ordered structure (D-Ti_3_C_2_T_*x*_/(ANF/PVA)) are then prepared by freeze-drying and hot pressing. Constructing a directionally porous structure enables the highly conductive Ti_3_C_2_T_*x*_ nanosheets to be wrapped on the directionally porous D-ANF/PVA framework in order arrangement and overlapped with each other. And the hot pressing process effectively reduces the layer spacing between the stacked wavy D-ANF/PVA, to form a large number of Ti_3_C_2_T_*x*_-Ti_3_C_2_T_*x*_ continuous conductive paths, which significantly improves the conductivity of the D-Ti_3_C_2_T_*x*_/(ANF/PVA) EMI shielding composite film. When the amount of Ti_3_C_2_T_*x*_ is 80 wt%, the EMI shielding effectiveness (EMI SE) and specific SE (SSE/*t*) of D-Ti_3_C_2_T_*x*_/(ANF/PVA) EMI shielding composite film achieve 70 dB and 13790 dB·cm^2^·g^−1^ (thickness and density of 120 *μ*m and 0.423 g·cm^−3^), far superior to random-structured Ti_3_C_2_T_*x*_/(ANF/PVA) (R-Ti_3_C_2_T_*x*_/(ANF/PVA)) composite film (46 dB and 9062 dB·cm^2^·g^−1^, respectively) *via* blending-freeze-drying followed by hot pressing technology. Meanwhile, the D-Ti_3_C_2_T_*x*_/(ANF/PVA) EMI shielding composite film possesses excellent flexibility and foldability.

## 1. Introduction

With the rapid development and widespread use of flexible wearable electronic equipment and 5G communication technology, the resulting electromagnetic radiation and electromagnetic pollution are increasing [1], which not only interferes with the normal operation of sophisticated electronic devices but also threatens the health of surrounding people [2–4]. In recent years, the rapid development of electromagnetic interference (EMI) shielding materials has made the disadvantages of traditional metal materials increasingly apparent [5–7]. An urgent need has been raised for materials to be ultrathin and flexible and to have excellent EMI shielding effectiveness (EMI SE), mechanical properties, and corrosion resistance [8–10]. Therefore, multifunctional polymer-based EMI shielding composite films have become one of the current research hotspots in the field of EMI shielding materials [11–13].

Two-dimensional transition metal carbide/nitride Ti_3_C_2_T_*x*_ is widely used in the field of EMI shielding due to its excellent conductivity [14–16]. Moreover, it has abundant surface functional groups, excellent water dispersibility, and film-forming performance and can be used to prepare polymer-based EMI shielding composite films with excellent comprehensive properties [17–19]. At present, most researchers have used blending or layer-by-layer alternating methods to prepare Ti_3_C_2_T_*x*_/polymer EMI shielding composite films [20–23]. When preparing Ti_3_C_2_T_*x*_/polymer EMI shielding composite films by blending, highly conductive Ti_3_C_2_T_*x*_ is disorderly distributed in the insulating polymer matrix [24–26]. Usually, a large amount of Ti_3_C_2_T_*x*_ is required to build the continuous and efficient conductive network, which will affect the processing behaviors and mechanical properties of the composite films [27]. The layer-by-layer alternating method is to arrange the polymer matrix and the highly conductive Ti_3_C_2_T_*x*_ alternately to prepare Ti_3_C_2_T_*x*_/polymer EMI shielding composite films. The mechanical framing effect exerted by the polymer layer can prevent the nanoscale “sawtooth” cracks in the Ti_3_C_2_T_*x*_ layer from growing to the entire composite films, giving excellent mechanical properties [28]. However, the multilayer structure destroys the Ti_3_C_2_T_*x*_ conductive network to a certain extent, causing the electrical conductivity (*σ*) and EMI SE of the composite films to decrease. The above two general preparation methods both have the problem of disordered conductive networks. It is urgent to explore EMI shielding composite films with an ordered conductive network to improve their *σ* [29] and at the same time to enhance internal multiple reflections, in order to achieve efficient improvement of EMI shielding performance with a low amount of Ti_3_C_2_T_*x*_.

Constructing an orderly conductive network of the EMI shielding composite films can rely on the preparation of the orderly porous structure aerogel in the early stage [30]. Directional freeze-drying is a novel technology that uses the directional growth of ice crystals to construct directionally porous structures [31–33]. It has the characteristics of simple and easy operation, no chemical reaction, and no by-products. Studies have shown that the obtained aerogel with a regularly directionally porous structure can form multiple reflection losses on electromagnetic waves and help to achieve excellent EMI shielding performances [34, 35]. Zhao et al. [36] prepared Ti_3_C_2_T_*x*_/reduced graphene oxide (Ti_3_C_2_T_*x*_/rGO) hybrid aerogels with a directionally porous structure using hydrothermal-assisted self-assembly and directional freeze-drying. When the thickness was 2 mm and the amount of Ti_3_C_2_T_*x*_ was 0.74 vol%, the EMI SE of the Ti_3_C_2_T_*x*_/rGO hybrid aerogel at the X band was as high as 56 dB. Wu et al. [37] prepared Ti_3_C_2_T_*x*_/SA aerogel by blending Ti_3_C_2_T_*x*_ and sodium alginate (SA) *via* directional freeze-drying and then coated a thin layer of polydimethylsiloxane (PDMS) on the surface of Ti_3_C_2_T_*x*_/SA aerogel by dip coating, improving the stability and durability of the porous structure. When the amount of Ti_3_C_2_T_*x*_ was 95 wt%, the successful construction of the 3D conductive network endowed Ti_3_C_2_T_*x*_/SA aerogels excellent *σ* (2211 S/m) and EMI SE (70.5 dB). However, when simple blending-directional freeze-drying is used to prepare aerogels, the highly conductive Ti_3_C_2_T_*x*_ is mixed with the insulating polymer matrix disorderly. It is still difficult to build a continuous and efficient conductive network and hard to achieve excellent EMI shielding performances with a low amount of Ti_3_C_2_T_*x*_ [38].

High EMI shielding performances of polymer-based composites with a low amount of Ti_3_C_2_T_*x*_ can be viable if a polymer framework with a directionally porous structure is prepared by directional freeze-drying, followed by the vacuum-assisted impregnation process to wrap the highly conductive Ti_3_C_2_T_*x*_ onto the polymer framework [39]. Polyvinyl alcohol (PVA) is an ideal polymer matrix that can be used for directional freeze-drying, but the mechanical properties of PVA aerogels are relatively poor, so that it is difficult to ensure structural stability during ultrasonication and vacuum-assisted impregnation [40, 41]. Aramid nanofibers (ANFs) are organic nanofibers with excellent characteristics such as lightweight, high strength, and high temperature resistance [42]. Incorporating high-performance ANFs into PVA is expected to significantly enhance the mechanical properties of PVA aerogels. In addition, the presence of a large amount of air in the pores of the aerogel with a directionally porous structure will make it difficult for Ti_3_C_2_T_*x*_ wrapped on the polymer framework to contact each other and difficult to form the efficient Ti_3_C_2_T_*x*_ conductive network, which would exhibit low conductivity. If the aerogels are pressed into films by hot pressing, the layer spacing between the stacked wavy polymer framework can be greatly reduced, facilitating the Ti_3_C_2_T_*x*_ wrapped on the polymer framework to contact each other. Then, a large number of efficient Ti_3_C_2_T_*x*_ conductive paths are expected to be formed to significantly improve *σ* and EMI SE of the composite films.

Herein, directional freeze-drying is used to prepare ANF/PVA aerogels with a directionally porous structure (D-ANF/PVA), and then, Ti_3_C_2_T_*x*_ dispersion is fully and uniformly immersed into D-ANF/PVA aerogels *via* ultrasonication and vacuum-assisted impregnation. Ti_3_C_2_T_*x*_/(ANF/PVA) aerogels with a directionally porous structure (D-Ti_3_C_2_T_*x*_/(ANF/PVA)) are obtained by freeze-drying, and then, the directionally ordered D-Ti_3_C_2_T_*x*_/(ANF/PVA) EMI shielding composite films are prepared by hot pressing. The effects of the amount of Ti_3_C_2_T_*x*_ on *σ*, EMI SE, and mechanical properties of the D-Ti_3_C_2_T_*x*_/(ANF/PVA) EMI shielding composite films are discussed in detail.

## 2. Results and Discussion

**Figure 1:**
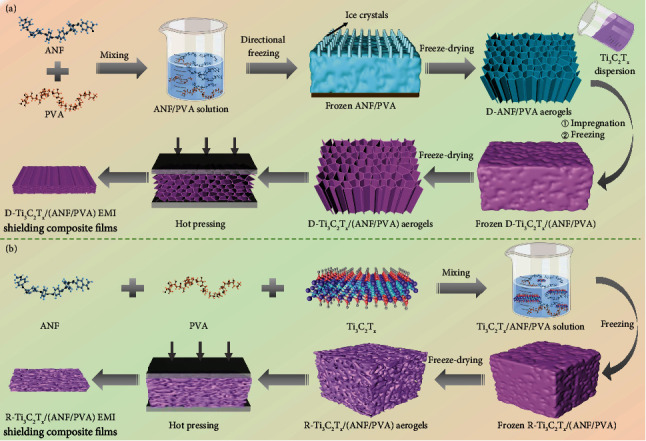
Schematic diagrams for the preparation of D-Ti_3_C_2_T_*x*_/(ANF/PVA) (a) and R-Ti_3_C_2_T_*x*_/(ANF/PVA) (b) EMI shielding composite films.

The process of preparing D-Ti_3_C_2_T_*x*_/(ANF/PVA) and R-Ti_3_C_2_T_*x*_/(ANF/PVA) EMI shielding composite films is shown in Figure 1. The directional freeze-drying technology is utilized to prepare the D-ANF/PVA aerogel, and the Ti_3_C_2_T_*x*_ dispersion is fully and uniformly immersed into the D-ANF/PVA aerogel *via* ultrasonication and vacuum-assisted impregnation. The D-Ti_3_C_2_T_*x*_/(ANF/PVA) EMI shielding composite films with a directionally ordered structure are prepared by freeze-drying and hot pressing. The R-Ti_3_C_2_T_*x*_/(ANF/PVA) EMI shielding composite films are prepared by blending-freeze drying-hot pressing technology. The experimental details can be found in Materials and Methods.

**Figure 2:**
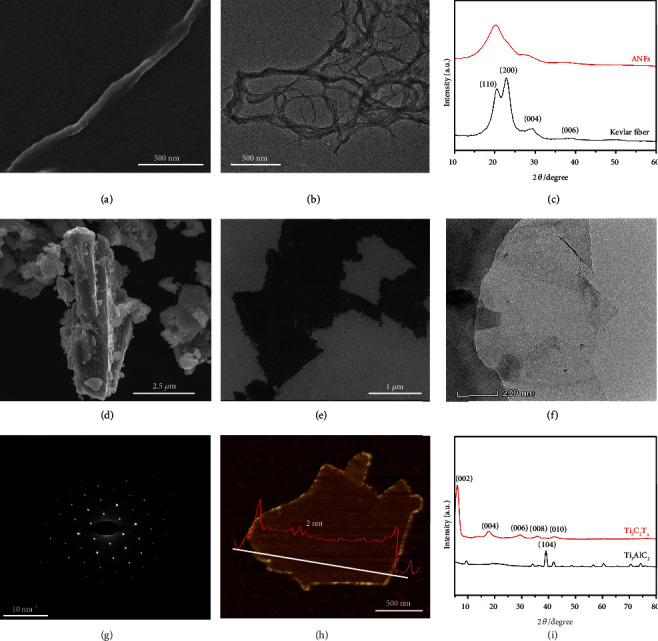
SEM (a) and TEM (b) images of ANFs; XRD spectra of Kevlar fiber and ANFs (c); SEM images of Ti_3_AlC_2_ (d) and Ti_3_C_2_T_*x*_ (e); TEM image (f) and SAED pattern (g) of Ti_3_C_2_T_*x*_; AFM image of Ti_3_C_2_T_*x*_ (h); XRD spectra of Ti_3_AlC_2_ and Ti_3_C_2_T_*x*_ (i).

From Figures 2(a) and 2(b), the ANF fibers are slender threads, with length of about 5~10 *μ*m and diameter of about 40~50 nm, and they overlap each other. This is attributed to the fact that there are amide bonds between the molecular chains of Kevlar fibers, and strong hydrogen bonds are formed between the molecular chains. After being treated with strong base KOH, the hydrogen in the amide group undergoes deprotonation; a large number of hydrogen bonds are destroyed and gradually dissociated [43]. The electrostatic repulsion between the molecular chains further promotes the dissociation of the Kevlar fiber and gradually reduces the Kevlar fiber size. However, the entanglement of the molecular chains causes the benzene rings to stack on each other to generate *π*‐*π* conjugated interaction force, which prevents the Kevlar fiber from disintegrating completely, thus forming nanofibrous ANFs.  Figure 2(c) shows the XRD spectra of Kevlar fibers and ANFs. The diffraction peaks of Kevlar fiber at 21°, 23°, 28°, and 39° correspond to (110), (200), (004), and (006) crystal planes, respectively, mainly due to the regular arrangement of the molecular chain structure inside the Kevlar fiber and the abundant hydrogen bond interactions that give rise to a higher degree of crystallinity [44]. The intensity of the crystallization peak in ANFs is greatly reduced, only with a broad diffraction peak near 20°, indicating that the crystal structure of Kevlar fiber is dissociated and the hydrogen bond between molecular chains is broken [45]. Ti_3_AlC_2_ has a compact layered structure (Figure 2(d)) [46]. After etching with hydrofluoric acid generated *in situ* by lithium fluoride and hydrochloric acid, Ti_3_C_2_T_*x*_ nanosheets with a two-dimensional lamella structure are obtained (Figure 2(e)). Ti_3_C_2_T_*x*_ nanosheets are highly transparent under electron irradiation (Figure 2(f)), indicating that they are very thin and have a clear surface without impurities [47]. The corresponding selected area electron diffraction (SAED) (Figure 2(g)) shows that the Ti_3_C_2_T_*x*_ nanosheets have a typical hexagonal crystal structure. From the AFM image (Figure 2(h)), the Ti_3_C_2_T_*x*_ nanosheets are regular in shape, with radial size of about 1.4 *μ*m and thickness of about 2 nm. The XRD spectrum of Ti_3_C_2_T_*x*_ nanosheets (Figure 2(i)) shows sharp diffraction peaks at 6° and weaker diffraction peaks at 13°, 19°, 26°, and 32°, corresponding to (002), (004), (006), (008), and (010) crystal planes, respectively [48, 49]. The peak at 39° for the (104) crystal plane in Ti_3_AlC_2_ disappears, and the peak intensity of the (002) crystal plane is much higher than others [50, 51]. The above characterizations indicate the successful preparation of few-layered Ti_3_C_2_T_*x*_.

**Figure 3:**
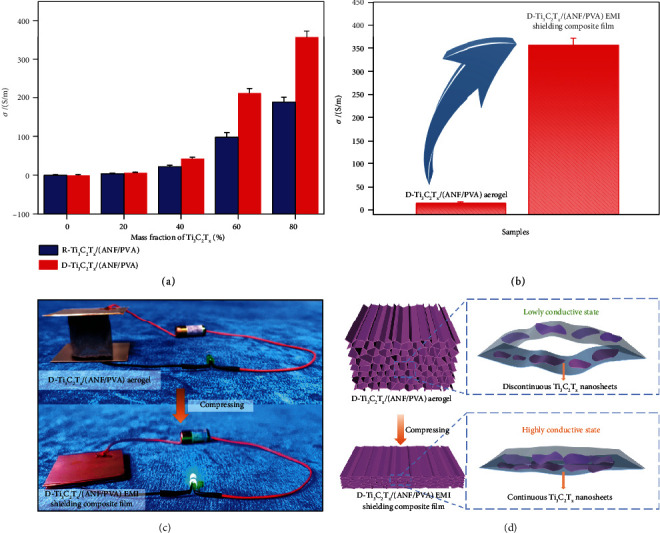
*σ* of R-Ti_3_C_2_T_*x*_/(ANF/PVA) and D-Ti_3_C_2_T_*x*_/(ANF/PVA) EMI shielding composite films (a); *σ* of D-Ti_3_C_2_T_*x*_/(ANF/PVA) aerogel and D-Ti_3_C_2_T_*x*_/(ANF/PVA) EMI shielding composite film (b); photographs of D-Ti_3_C_2_T_*x*_/(ANF/PVA) aerogel and D-Ti_3_C_2_T_*x*_/(ANF/PVA) EMI shielding composite film integrated into LED bulb circuits (c); schematic illustration for the conductive mechanism of D-Ti_3_C_2_T_*x*_/(ANF/PVA) aerogel and D-Ti_3_C_2_T_*x*_/(ANF/PVA) EMI shielding composite film (d).

**Figure 4:**
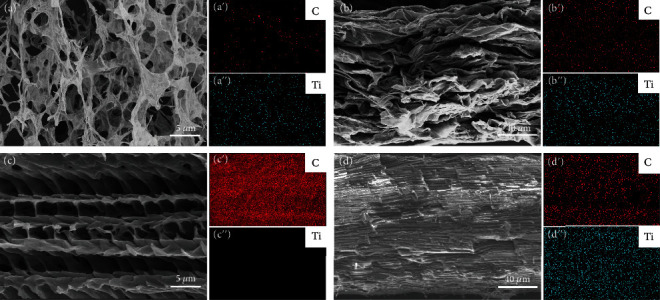
SEM images for cross-section of R-Ti_3_C_2_T_*x*_/(ANF/PVA) aerogel (a) with EDS element distribution diagrams of C (a') and Ti (a”) elements, respectively; SEM images for cross-section of R-Ti_3_C_2_T_*x*_/(ANF/PVA) EMI shielding composite film (b) with EDS element distribution diagrams of C (b') and Ti (b”) elements, respectively; SEM images for cross-section of D-ANF/PVA aerogel (c) with EDS element distribution diagrams of C (c') and Ti (c”) elements, respectively; SEM images for cross-section of D-Ti_3_C_2_T_*x*_/(ANF/PVA) EMI shielding composite film (d) with EDS element distribution diagrams of C (d') and Ti (d”) elements, respectively.

Figure 3(a) shows *σ* of R-Ti_3_C_2_T_*x*_/(ANF/PVA) and D-Ti_3_C_2_T_*x*_/(ANF/PVA) EMI shielding composite films. With the increase in the amount of Ti_3_C_2_T_*x*_, *σ* of R-Ti_3_C_2_T_*x*_/(ANF/PVA) and D-Ti_3_C_2_T_*x*_/(ANF/PVA) EMI shielding composite films show the trend of rapid increase. This is because the intrinsic conductivity of Ti_3_C_2_T_*x*_ is very high. With the increase in the amount of Ti_3_C_2_T_*x*_, the conductive networks inside the composite films are gradually improved to be complete, leading the conductivity of the composite films to increase with the increase in the amount of Ti_3_C_2_T_*x*_ [52, 53]. When the amount of Ti_3_C_2_T_*x*_ is 80 wt%, *σ* of the R-Ti_3_C_2_T_*x*_/(ANF/PVA) EMI shielding composite film increases to 188.7 S/m, and that of the D-Ti_3_C_2_T_*x*_/(ANF/PVA) EMI shielding composite film is as high as 357.1 S/m, much higher than that of the R-Ti_3_C_2_T_*x*_/(ANF/PVA) EMI shielding composite film. This is because Ti_3_C_2_T_*x*_ is disorderly distributed in the R-Ti_3_C_2_T_*x*_/(ANF/PVA) EMI shielding composite film, and ANFs and PVA are interspersed between the conductive Ti_3_C_2_T_*x*_ layers, making it difficult to form an efficient conductive network (Figures 4(a) and 4(b”) and Figure S2(a)). The D-ANF/PVA aerogel prepared by directional freeze-drying has a neat and directionally porous structure (Figures 4(c)–4(c”)). In the directional freezing process, the water in the ANF/PVA dispersion is affected by the supercooling provided by the cold source to form ice crystals, which grow vertically upwards along the freezing gradient direction, promoting the orderly arrangement of ANF/PVA along the growth direction of the ice crystals. During the low-pressure drying process, the ice crystals sublime to obtain D-ANF/PVA aerogel with a directionally porous structure. After pouring the Ti_3_C_2_T_*x*_ dispersion, highly conductive Ti_3_C_2_T_*x*_ nanosheets are neatly and orderly wrapped onto the outer surfaces of the D-ANF/PVA aerogel and the inner walls of the through-hole, forming a directionally ordered continuous 3D conductive network (Figure S1(a-a”)). Furthermore, the hot pressing process compresses the directional hole wall between the D-Ti_3_C_2_T_*x*_/(ANF/PVA) aerogel (Figures 4(d)–4(d”) and Figure S2(b)), and the Ti_3_C_2_T_*x*_ nanosheets in the through-hole are in efficient contact. The D-ANF/PVA aerogel contains C element, while D-Ti_3_C_2_T_*x*_/(ANF/PVA) aerogel and corresponding composite film contain not only C element but also Ti element. This is mainly due to the successful introduction of Ti_3_C_2_T_*x*_. Therefore, a large number of continuous and efficient Ti_3_C_2_T_*x*_ conductive paths are formed, and *σ* of the D-Ti_3_C_2_T_*x*_/(ANF/PVA) EMI shielding composite films is significantly improved [54]. As shown in Figures 3(b)–3(d), *σ* of D-Ti_3_C_2_T_*x*_/(ANF/PVA) aerogel is only about 15.3 S/m, which fails to make the light-emitting diode (LED) bulb light up, showing low conductivity. *σ* of the D-Ti_3_C_2_T_*x*_/(ANF/PVA) EMI shielding composite film formed by hot pressing is as high as about 357.1 S/m, which lights the LED bulb, exhibiting high conductivity.

**Figure 5:**
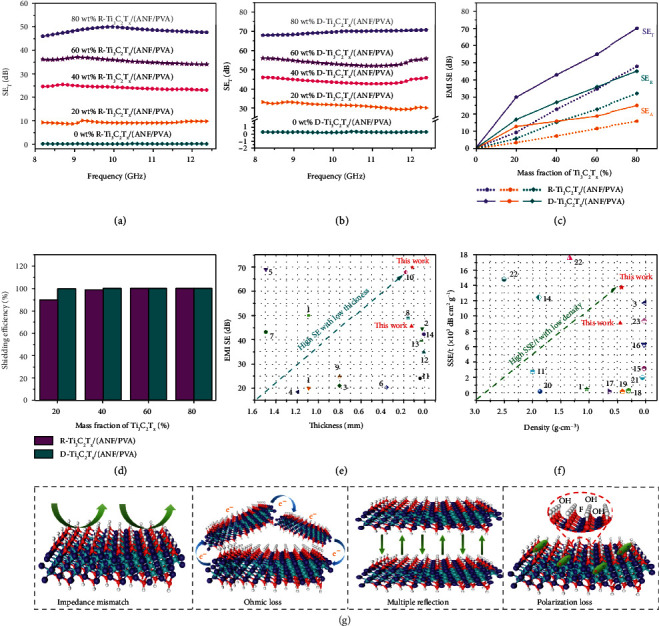
SE_T_ of R-Ti_3_C_2_T_*x*_/(ANF/PVA) (a) and D-Ti_3_C_2_T_*x*_/(ANF/PVA) (b) EMI shielding composite films; comparison of SE_T_, SE_A_, and SE_R_ for R-Ti_3_C_2_T_*x*_/(ANF/PVA) and D-Ti_3_C_2_T_*x*_/(ANF/PVA) EMI shielding composite films (c); shielding efficiency of R-Ti_3_C_2_T_*x*_/(ANF/PVA) and D-Ti_3_C_2_T_*x*_/(ANF/PVA) EMI shielding composite films (d); EMI SE *vs.* thickness (e) and SSE/*tvs.* density (f) of R-Ti_3_C_2_T_*x*_/(ANF/PVA) and D-Ti_3_C_2_T_*x*_/(ANF/PVA) EMI shielding composite films compared with other works, respectively; schematic diagram for EMI shielding mechanism of D-Ti_3_C_2_T_*x*_/(ANF/PVA) composite films (g).

Figures 5(a) and 5(b) and Figures S3 and S4 show the total shielding effectiveness (SE_T_), absorption shielding effectiveness (SE_A_), and reflection shielding effectiveness (SE_R_) of R-Ti_3_C_2_T_*x*_/(ANF/PVA) and D-Ti_3_C_2_T_*x*_/(ANF/PVA) EMI shielding composite films at the X band, respectively. The SE_T_, SE_A_, and SE_R_ of ANF/PVA composite films are all very low, about 0.4 dB, 0.3 dB, and 0.1 dB, respectively. As the amount of Ti_3_C_2_T_*x*_ increases, the SE_T_, SE_A_, and SE_R_ of R-Ti_3_C_2_T_*x*_/(ANF/PVA) and D-Ti_3_C_2_T_*x*_/(ANF/PVA) EMI shielding composite films increase significantly. When the amount of Ti_3_C_2_T_*x*_ is 80 wt%, the SE_T_, SE_A_, and SE_R_ of the D-Ti_3_C_2_T_*x*_/(ANF/PVA) EMI shielding composite film reach 70 dB, 24 dB, and 46 dB, respectively, much higher than SE_T_ (46 dB), SE_A_ (16 dB), and SE_R_ (30 dB) of the R-Ti_3_C_2_T_*x*_/(ANF/PVA) EMI shielding composite film (Figure 5(c)). Meanwhile, D-Ti_3_C_2_T_*x*_/(ANF/PVA) EMI shielding composite film has higher EMI shielding efficiency. When the amount of Ti_3_C_2_T_*x*_ is 80 wt%, D-Ti_3_C_2_T_*x*_/(ANF/PVA) EMI shielding composite film is capable of blocking 99.99999% of electromagnetic wave radiation, far better than that of R-Ti_3_C_2_T_*x*_/(ANF/PVA) EMI shielding composite film, which could block 99.997% of electromagnetic wave radiation (Figure 5(d)).

Poor EMI shielding performance of the R-Ti_3_C_2_T_*x*_/(ANF/PVA) composite films is mainly attributed to the disorderly arrangement of the Ti_3_C_2_T_*x*_ nanosheets inside. Contact between Ti_3_C_2_T_*x*_ as well as formation of an effective Ti_3_C_2_T_*x*_ conductive network is difficult, making *σ* low. In addition, the disorderly and chaotic arrangement of Ti_3_C_2_T_*x*_ nanosheets in the R-Ti_3_C_2_T_*x*_/(ANF/PVA) composite film also results in less internal multiple reflection and scattering of electromagnetic waves.

As shown in Figure S5, we performed 500 and 1000 folding cycles of the D-Ti_3_C_2_T_*x*_/(ANF/PVA) composite film with the mass fraction of Ti_3_C_2_T_*x*_ of 80 wt%. It can be seen that its EMI shielding effectiveness has almost no change, which shows that the EMI shielding performance of composite film is stable and repeatable.

Superior EMI shielding performance of D-Ti_3_C_2_T_*x*_/(ANF/PVA) composite films is mainly due to its multiple EMI shielding effects. Firstly, the construction of a directionally ordered structure and the adoption of the hot pressing process give the D-Ti_3_C_2_T_*x*_/(ANF/PVA) EMI shielding composite films higher *σ*, which is more different from the *σ* of air, causing greater impedance mismatch. Therefore, when external electromagnetic waves are incident on the surface of the D-Ti_3_C_2_T_*x*_/(ANF/PVA) EMI shielding composite films, a large proportion of them is immediately reflected back into the air [55]. Secondly, due to the excellent *σ* of the D-Ti_3_C_2_T_*x*_/(ANF/PVA) EMI shielding composite films, microcurrents are generated by electromagnetic waves through charge carriers, which enhances the ohmic loss of electromagnetic waves and reduces the energy of electromagnetic waves [56]. Thirdly, a large amount of internal multiple reflection and scattering for remaining electromagnetic waves occur between the neatly arranged and parallel Ti_3_C_2_T_*x*_ layers, where the energy of electromagnetic waves is converted into heat in the form of microcurrent, thus greatly enhancing the loss of electromagnetic wave energy [57], further improving the electromagnetic wave absorption ability of the D-Ti_3_C_2_T_*x*_/(ANF/PVA) EMI shielding composite films. Lastly, the electromagnetic waves also experience polarization loss with the functional groups (-OH, -F, etc.) on the surface of Ti_3_C_2_T_*x*_ [58]. Therefore, D-Ti_3_C_2_T_*x*_/(ANF/PVA) EMI shielding composite films have higher EMI SE than that of R-Ti_3_C_2_T_*x*_/(ANF/PVA) EMI shielding composite films. Therefore, the neat and orderly D-Ti_3_C_2_T_*x*_/(ANF/PVA) EMI shielding composite films realize the reflection of electromagnetic waves, internal multiple reflections, and absorption loss, and only a very small amount of electromagnetic waves could pass through the D-Ti_3_C_2_T_*x*_/(ANF/PVA) EMI shielding composite films, which can greatly reduce the electromagnetic pollution to the environment as well as the harm to human health (Figure 5(g)).

In order to further prove the superiority of the EMI shielding performance of the D-Ti_3_C_2_T_*x*_/(ANF/PVA) composite films, the obtained EMI shielding performances in this work have been compared with other polymer-based materials reported in literatures (Tables S2 and S3). Figures 5(e) and 5(f) show the comparisons of the EMI SE *vs.* thickness and specific shielding effectiveness (SSE/*t*) *vs.* density, respectively. With the thickness of 120 *μ*m and the density of 0.423 g·cm^−3^, the D-Ti_3_C_2_T_*x*_/(ANF/PVA) EMI shielding composite film achieves excellent EMI SE (70 dB) and SSE/*t* (13790 dB·cm^2^·g^−1^), much higher than those of R-Ti_3_C_2_T_*x*_/(ANF/PVA) EMI shielding composite film (46 dB and 9062 dB·cm^2^·g^−1^, respectively) and similar materials reported in literatures.

**Figure 6:**
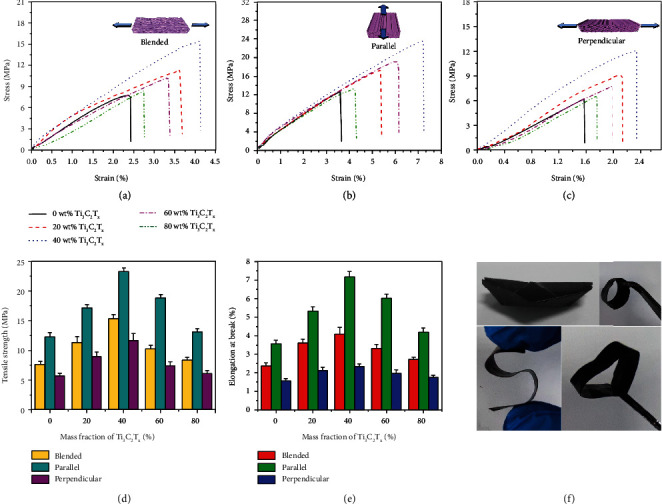
Stress-strain curves of the R-Ti_3_C_2_T_*x*_/(ANF/PVA) EMI shielding composite films (a) and D-Ti_3_C_2_T_*x*_/(ANF/PVA) EMI shielding composite films stretched parallel (b) and perpendicular (c) to the directional freezing direction; tensile strength (d) and elongation at break (e) of R-Ti_3_C_2_T_*x*_/(ANF/PVA) and D-Ti_3_C_2_T_*x*_/(ANF/PVA) EMI shielding composite films; photographs of D-Ti_3_C_2_T_*x*_/(ANF/PVA) EMI shielding composite films (f).

Figures 6(a)–6(c) show the stress-strain curves of R-Ti_3_C_2_T_*x*_/(ANF/PVA) and D-Ti_3_C_2_T_*x*_/(ANF/PVA) EMI shielding composite films, and the corresponding tensile strength and elongation at break are shown in Figures 6(d) and 6(e). The tensile properties of the D-Ti_3_C_2_T_*x*_/(ANF/PVA) EMI shielding composite film stretched parallel to the directional freezing direction is much higher than that of the R-Ti_3_C_2_T_*x*_/(ANF/PVA) EMI shielding composite film. When the amount of Ti_3_C_2_T_*x*_ is 80 wt%, the tensile strength and elongation at break of the R-Ti_3_C_2_T_*x*_/(ANF/PVA) EMI shielding composite film are 8.4 MPa and 2.7%, respectively. At the same amount of Ti_3_C_2_T_*x*_, the tensile strength and elongation at break of the D-Ti_3_C_2_T_*x*_/(ANF/PVA) EMI shielding composite film stretched parallel to the directional freezing direction are 13.1 MPa and 4.2%, respectively. This is because the distribution of Ti_3_C_2_T_*x*_ in the R-Ti_3_C_2_T_*x*_/(ANF/PVA) EMI shielding composite film is relatively chaotic and disorderly, and more stress concentration points are easily generated when subjected to external forces. For D-Ti_3_C_2_T_*x*_/(ANF/PVA) EMI shielding composite films, the structure is directionally ordered parallel to the directional freezing direction, and its stress distribution is uniform, giving it high tensile strength and elongation at break [59, 60]. In addition, when the amount of Ti_3_C_2_T_*x*_ is 80 wt%, the tensile strength and elongation at break of the D-Ti_3_C_2_T_*x*_/(ANF/PVA) EMI shielding composite film stretched perpendicular to the directional freezing direction are 6.1 MPa and 1.7%, respectively, lower than those of D-Ti_3_C_2_T_*x*_/(ANF/PVA) EMI shielding composite film stretched parallel to the directional freezing direction, indicating that the mechanical properties of the D-Ti_3_C_2_T_*x*_/(ANF/PVA) EMI shielding composite films are anisotropic. This may be attributed to the fact that the structural strength perpendicular to the directional freezing direction is relatively weak, and the weaker intermittent connection will break under lower tensile strength. In contrast, the structure parallel to the directional freezing direction is relatively complete, with fewer internal defects [61, 62]. Moreover, it should be noted that the D-Ti_3_C_2_T_*x*_/(ANF/PVA) EMI shielding composite film can be folded into a paper boat and bent into circle, “S,” and heart shapes (Figure 6(e)), indicating that it has excellent flexibility and foldability.

## 3. Conclusions

Construction of the directionally porous structure enables the highly conductive Ti_3_C_2_T_*x*_ nanosheets to be wrapped on the directionally ordered D-ANF/PVA framework in orderly arrangement and overlapped with each other, achieving a more efficient and complete conductive network with the same amount of Ti_3_C_2_T_*x*_. The hot pressing would process greatly reduce the layer spacing between the stacked wavy D-ANF/PVA and enable a large number of Ti_3_C_2_T_*x*_ nanosheets wrapped on the D-ANF/PVA framework to efficiently contact. A large number of Ti_3_C_2_T_*x*_-Ti_3_C_2_T_*x*_ continuous conductive paths are formed, which significantly improve *σ* of D-Ti_3_C_2_T_*x*_/(ANF/PVA) EMI shielding composite films. When the amount of Ti_3_C_2_T_*x*_ is 80 wt%, EMI SE and SSE/*t* of D-Ti_3_C_2_T_*x*_/(ANF/PVA) EMI shielding composite film achieve 70 dB and 13790 dB·cm^2^·g^−1^ (thickness and density of 120 *μ*m and 0.423 g·cm^−3^), far superior to R-Ti_3_C_2_T_*x*_/(ANF/PVA) EMI shielding composite film (46 dB and 9062 dB·cm^2^·g^−1^, respectively) prepared by blending-freeze drying followed by hot pressing technology. The mechanical properties of the D-Ti_3_C_2_T_*x*_/(ANF/PVA) EMI shielding composite films are anisotropic. When the amount of Ti_3_C_2_T_*x*_ is 80 wt%, the tensile strength and elongation at break stretched parallel to the directional freezing direction are 13.1 MPa and 4.2%, respectively, significantly better than the tensile strength (8.4 MPa) and elongation at break (2.7%) of the R-Ti_3_C_2_T_*x*_/(ANF/PVA) EMI shielding composite film. At the same time, the D-Ti_3_C_2_T_*x*_/(ANF/PVA) EMI shielding composite film possesses excellent flexibility and foldability. It can be folded into a paper boat and bent into circle, “S,” and heart shapes.

## 4. Materials and Methods

### 4.1. Preparation of D-ANF/PVA Aerogels

A certain amount of ANFs and PVA were added into 30 mL of deionized water, to obtain the ANF/PVA dispersion by ultrasonication. Then, the above mixtures were poured into a homemade mold (copper at the bottom and polytetrafluoroethylene around), followed by being placed into the liquid nitrogen to freeze. Then, the frozen products were placed in the vacuum freeze dryer for 72 hrs to obtain the D-ANF/PVA aerogels with directionally porous structures.

### 4.2. Fabrication of D-Ti_3_C_2_T_*x*_/(ANF/PVA) and R-Ti_3_C_2_T_*x*_/(ANF/PVA) EMI Shielding Composite Films

A certain amount of Ti_3_C_2_T_*x*_ (prepared based on the minimally intensive layer delamination (MILD) method in accordance with our previous works [63]) was dispersed in deionized water to obtain Ti_3_C_2_T_*x*_ aqueous dispersion. Subsequently, the obtained D-ANF/PVA aerogels were immersed in the Ti_3_C_2_T_*x*_ dispersion, and the Ti_3_C_2_T_*x*_ aqueous dispersion was fully filled into the ANF/PVA aerogels *via* ultrasonication and vacuum-assisted impregnation process. Then, the ANF/PVA aerogels impregnated with Ti_3_C_2_T_*x*_ aqueous dispersion were placed in liquid nitrogen and were then placed in the vacuum freeze dryer for 72 hrs to obtain D-Ti_3_C_2_T_*x*_/(ANF/PVA) aerogels. In this work, D-Ti_3_C_2_T_*x*_/(ANF/PVA) aerogels with Ti_3_C_2_T_*x*_ amount of 20 wt%, 40 wt%, 60 wt%, and 80 wt% were prepared, respectively. Finally, the D-Ti_3_C_2_T_*x*_/(ANF/PVA) aerogels were hot pressed at 50°C and 20 MPa for 10 min to obtain D-Ti_3_C_2_T_*x*_/(ANF/PVA) EMI shielding composite films.

For comparison, random-structured Ti_3_C_2_T_*x*_/(ANF/PVA) (R-Ti_3_C_2_T_*x*_/(ANF/PVA)) EMI shielding composite films were also prepared. A certain amount of Ti_3_C_2_T_*x*_, ANFs, and PVA were dispersed in deionized water, mixed uniformly by ultrasonication to obtain Ti_3_C_2_T_*x*_/(ANF/PVA) solution. Subsequently, the beaker containing Ti_3_C_2_T_*x*_/(ANF/PVA) solution was placed in liquid nitrogen, followed by staying in the vacuum freeze dryer for 72 hrs to obtain R-Ti_3_C_2_T_*x*_/(ANF/PVA) aerogels. Finally, the R-Ti_3_C_2_T_*x*_/(ANF/PVA) aerogels were hot pressed at 50°C and 20 MPa for 10 min to obtain R-Ti_3_C_2_T_*x*_/(ANF/PVA) EMI shielding composite films.

Specific experimental details such as raw materials and characterizations are provided in Supplementary Materials.

## Data Availability

The data in this paper cannot be shared at this time as the data also forms part of an ongoing study.

## Additional Points

*Highlights*. 1. Directionally porous structure is constructed so that the highly conductive Ti_3_C_2_T_x_ nanosheets are wrapped on the directionally ordered D-ANF/PVA framework in order arrangement and overlapped each other. 2. Hot-press process effectively reduces the layer spacing between the stacked wavy D-ANF/PVA, to form a large number of Ti_3_C_2_T_x_-Ti_3_C_2_T_x_ continuous conductive paths, which significantly improves the conductivity of the D-Ti_3_C_2_T_x_/(ANF/PVA) EMI shielding composite film. 3. When the amount of Ti_3_C_2_T_x_ is 80 wt%, the EMI SE and SSE/t of D-Ti_3_C_2_T_x_/(ANF/PVA) EMI shielding composite film achieve 70 dB and 13790 dB·cm^2^·g^−1^ (thickness and density of 120 *μ*m and 0.423 g·cm^−3^), far superior to R-Ti_3_C_2_T_x_/(ANF/PVA) EMI shielding composite film (46 dB and 9062 dB·cm^2^·g^−1^, respectively) by blending-freeze drying followed by hot pressing technology. 4. Mechanical properties of the D-Ti_3_C_2_T_x_/(ANF/PVA) EMI shielding composite film are anisotropic. When the amount of Ti_3_C_2_T_x_ is 80 wt%, the tensile strength of the D-Ti_3_C_2_T_x_/(ANF/PVA) EMI shielding composite film stretched parallel to the directional freezing direction are 13.1 MPa, significantly better than the tensile strength (8.4 MPa) of the R-Ti_3_C_2_T_x_/(ANF/PVA) EMI shielding composite film.

## Supplementary Materials

Supplementary Materials S1: experimental section. S1.1: main materials. S1.2: preparation of ANFs. S1.3: characterizations. S2: supporting results. FIGURE S1: SEM images for cross-section of D-Ti_3_C_2_T_*x*_/(ANF/PVA) aerogel (a) with EDS element distribution diagrams of C (a') and Ti (a”) elements, respectively. FIGURE S2: enlarged SEM images for cross-section of R-Ti_3_C_2_T_*x*_/(ANF/PVA) EMI shielding composite film (a) and D-Ti_3_C_2_T_*x*_/(ANF/PVA) EMI shielding composite film (b). FIGURE S3: SE_A_ (a) and SE_R_ (b) of R-Ti_3_C_2_T_*x*_/(ANF/PVA) EMI shielding composite films. FIGURE S4: SE_A_ (a) and SE_R_ (b) of D-Ti_3_C_2_T_*x*_/(ANF/PVA) EMI shielding composite films. FIGURE S5: the SE_T_ of D-Ti_3_C_2_T_*x*_/(ANF/PVA) composite film with the mass fraction of Ti_3_C_2_T_*x*_ of 80 wt% after 0, 500, and 1000 folding cycles. TABLE S1: shielding efficiency of R-Ti_3_C_2_T_*x*_/(ANF/PVA) and D-Ti_3_C_2_T_*x*_/(ANF/PVA) EMI shielding composite films. TABLE S2: comparison of EMI SE *vs.* thickness for different polymer matrix EMI shielding composites at X band. TABLE S3: comparison of SSE/*tvs.* density for different polymer matrix EMI shielding composites at X band.
