# Supplementary material for: Flexible Ti3C2Tx/(Aramid Nanofiber/PVA) Composite Films for Superior Electromagnetic Interference Shielding
Source: Research (Wash D C). 2022 Feb 2;2022:9780290. doi: 10.34133/2022/9780290 (PMC8832284; doi:10.34133/2022/9780290)
Supplement: Supplementary Materials — S1: experimental section. S1.1: main materials. S1.2: preparation of ANFs. S1.3: characterizations. S2: supporting results. FIGURE S1: SEM images for cross-section of D-Ti3C2Tx/(ANF/PVA) aerogel (a) with EDS element distribution diagrams of C (a') and Ti (a”) elements, respectively. FIGURE S2: enlarged SEM images for cross-section of R-Ti3C2Tx/(ANF/PVA) EMI shielding composite film (a) and D-Ti3C2Tx/(ANF/PVA) EMI shielding composite film (b). FIGURE S3: SEA (a) and SER (b) of R-Ti3C2Tx/(ANF/PVA) EMI shielding composite films. FIGURE S4: SEA (a) and SER (b) of D-Ti3C2Tx/(ANF/PVA) EMI shielding composite films. FIGURE S5: the SET of D-Ti3C2Tx/(ANF/PVA) composite film with the mass fraction of Ti3C2Tx of 80 wt% after 0, 500, and 1000 folding cycles. TABLE S1: shielding efficiency of R-Ti3C2Tx/(ANF/PVA) and D-Ti3C2Tx/(ANF/PVA) EMI shielding composite films. TABLE S2: comparison of EMI SE vs. thickness for different polymer matrix EMI shielding composites at X band. TABLE S3: comparison of SSE/tvs. density for different polymer matrix EMI shielding composites at X band. [file 9780290.f1.docx]

**Supplementary Materials**

**Flexible Ti_3_C_2_T_x_/(Aramid Nanofiber/PVA) Composite Films for Superior Electromagnetic Interference Shielding**

Yali Zhang, Zhonglei Ma, Kunpeng Ruan, Junwei Gu*

Shaanxi Key Laboratory of Macromolecular Science and Technology, School of Chemistry and Chemical Engineering, Northwestern Polytechnical University, Xi’ an, Shaanxi, 710072, P. R. China.

*Corresponding author: E-mail: gjw@nwpu.edu.cn & nwpugjw@163.com (J. Gu).

**S1. Experimental Section**

**S1.1 Main materials**

Polyvinyl alcohol (PVA, M_w_ ≈ 205,000, 99% hydrolyzed) was purchased from Guangzhou Jinhuada Chemical Reagent Co., Ltd. (Guangzhou, China). Ti_3_AlC_2_ powder (38 μm, 98% purity) was obtained from 11 Technology Co., Ltd. (Jilin, China). Concentrated hydrochloric acid (HCl) and lithium fluoride (LiF) were both purchased from Macklin Co., Ltd. (Shanghai, China). Commercial polyaramid fiber (Kevlar 49®) was purchased from Du Pont China Holding Co., Ltd. (Shanghai Branch, China). Potassium hydroxide (KOH) was supplied by Fu Yu Chemical Co., Ltd. (Tianjin, China). Dimethyl sulfoxide (DMSO) was purchased from Aladdin Co., Ltd. (Shanghai, China).

**S1.2** **Preparation of ANFs**

Chopped Kevlar fibers (2 g) and KOH (1.5 g) were added to 500 mL of DMSO and stirred at 30°C for 7 days, obtaining the reddish-brown solution accompanied with the dissociation of hydrogen bonds between the poly-p-phenylene terephthamide (PPTA) molecule chains. Then, 2500 mL of deionized water was added and mechanically stirred for 6 hrs at 200 rpm to make the flocculent ANFs fully precipitated. Afterward, the ANFs were washed by deionized water for several times in the Brinell funnel until the filtrate was neutral. After being washed, the ANF sludge was redispersed into the deionized water and treated with a homogenizer at 10,000 rpm for 10 min to obtain the stable ANF slurry.

**S1.3** **Characterizations**

X-ray diffraction (XRD) spectra of the samples were performed on a Shimadzu-7000 equipment (Shimadzu Co., Japan). Scanning electron microscope (SEM) images of the samples were collected on a VEGA3-LMH equipment (TESCAN Co., Czech Republic). Transmission electron microscopy (TEM) images of the samples were obtained on a Talos F200X/TEM microscope (FEI Co., USA) operated at 200 kV. Atomic force microscope (AFM) images of the samples were captured by a Dimension Fast Scan AFM (Bruker Co., USA). Electrical conductivities of the samples were collected by RTS-8 (Guangzhou Four Probes Technology Co., China). Electromagnetic interference shielding effectiveness (EMI SE) values of the samples were measured by an MS4644A Vector Network Analyzer instrument (Anritsu Co., Japan) using the wave-guide method at X-band according to ASTMD 5568-2008. Mechanical properties were tested by Instron Bluehill LE type microcomputer controlled electronic universal testing machine (Instron Co., USA) according to standard ISO1184-1983.

**S2.** **Supporting Results**


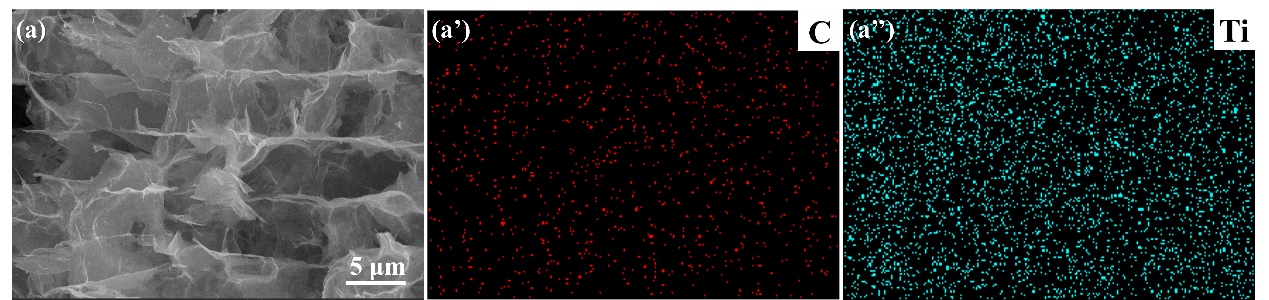


Figure S1: SEM images for cross section of D-Ti_3_C_2_T_x_/(ANF/PVA) aerogel (a) with EDS element distribution diagrams of C (a’) and Ti (a’’) elements, respectively.


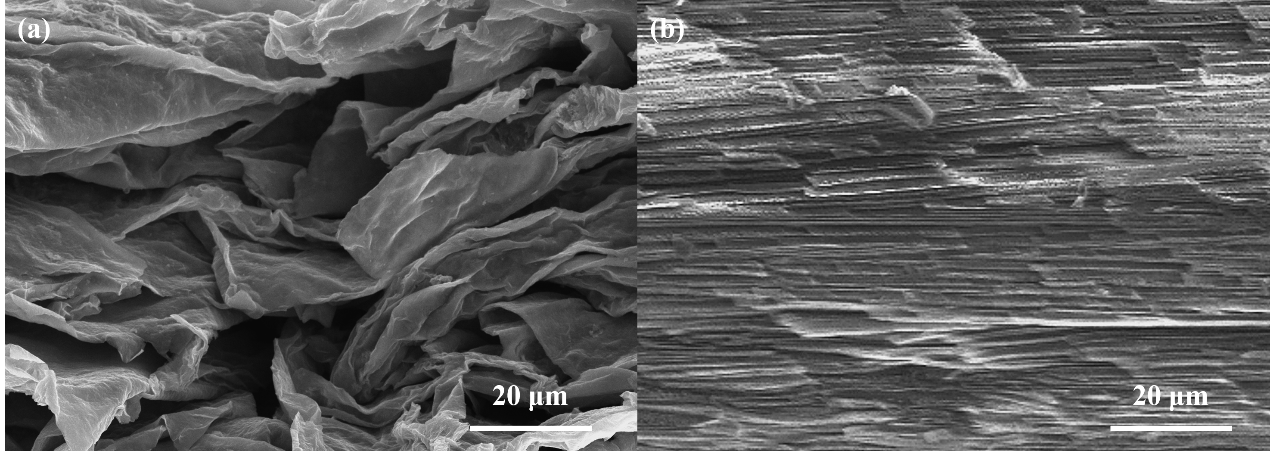


Figure S2: Enlarged SEM images for cross section of R-Ti_3_C_2_T_x_/(ANF/PVA) EMI shielding composite film (a) and D-Ti_3_C_2_T_x_/(ANF/PVA) EMI shielding composite film (b).


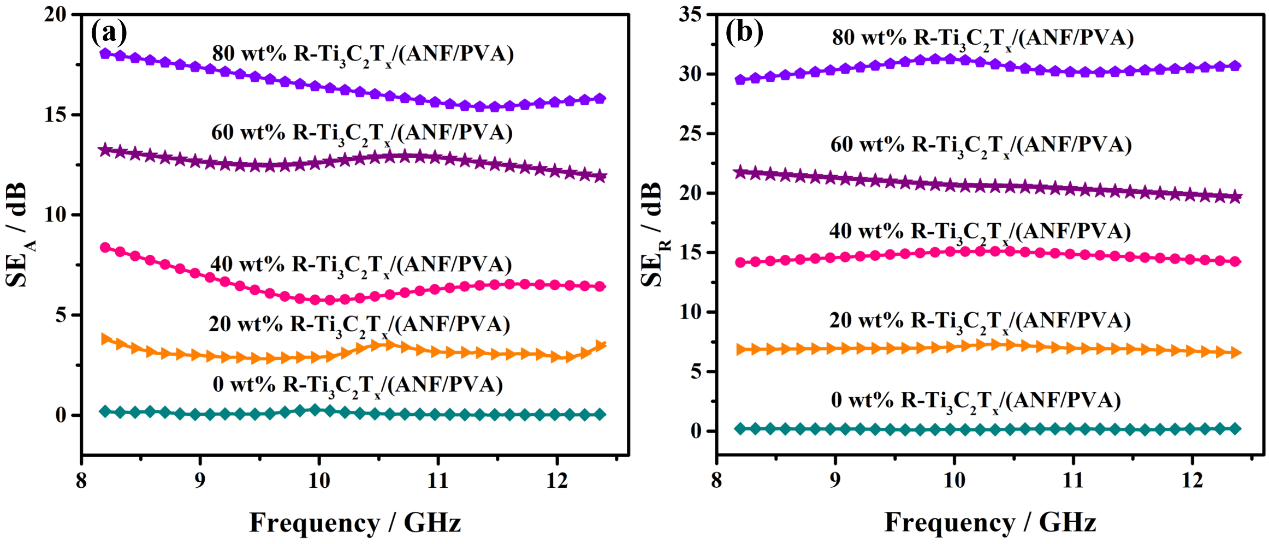


Figure S3: SE_A_ (a) and SE_R_ (b) of R-Ti_3_C_2_T_x_/(ANF/PVA) EMI shielding composite films.


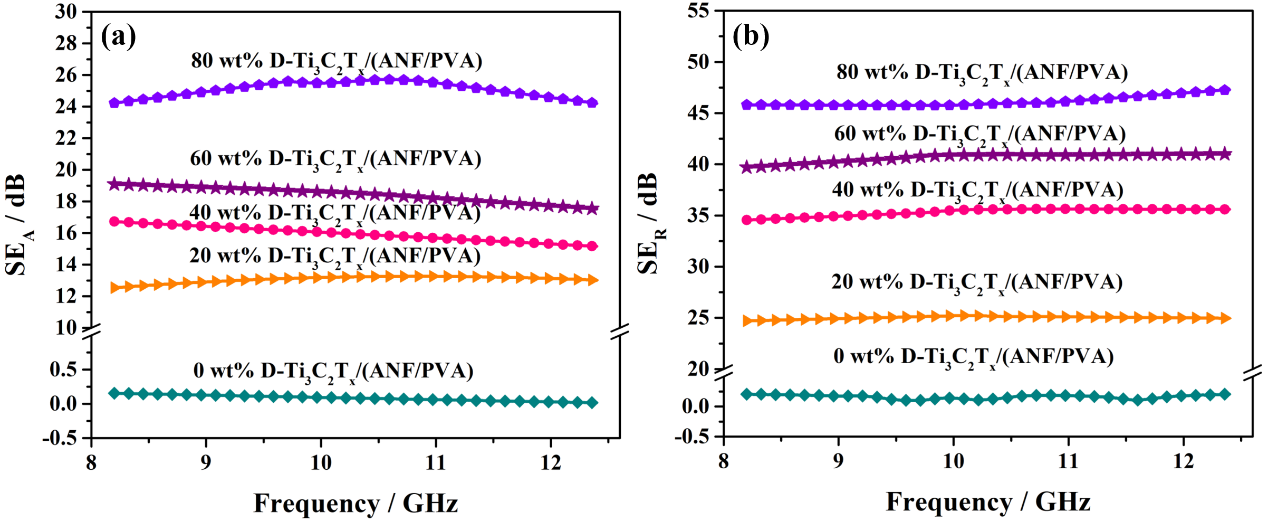


Figure S4: SE_A_ (a) and SE_R_ (b) of D-Ti_3_C_2_T_x_/(ANF/PVA) EMI shielding composite films.


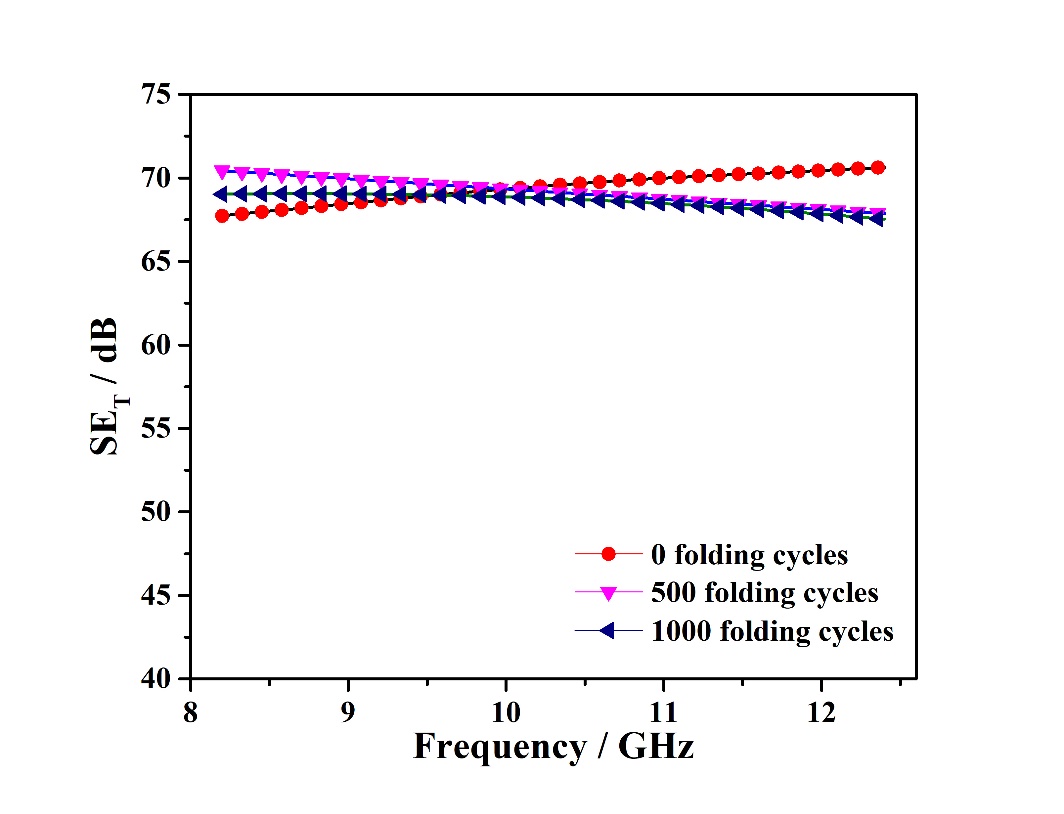


Figure S5: The SE_T_ of D-Ti_3_C_2_T_x_/(ANF/PVA) composite film with the mass fraction of Ti_3_C_2_T_x_ of 80 wt% after 0, 500 and 1000 folding cycles.

Table S1: Shielding efficiency of R-Ti_3_C_2_T_x_/(ANF/PVA) and D-Ti_3_C_2_T_x_/(ANF/PVA) EMI shielding composite films.

| **R-Ti_3_C_2_T_x_/(ANF/PVA)** | **Shielding efficiency (%)** | **D-Ti_3_C_2_T_x_/(ANF/PVA)** | **Shielding efficiency (%)** |
| --- | --- | --- | --- |
| 20 wt% Ti_3_C_2_T_x_ | 90 | 20 wt% Ti_3_C_2_T_x_ | 99.9 |
| 40 wt% Ti_3_C_2_T_x_ | 99 | 40 wt% Ti_3_C_2_T_x_ | 99.996 |
| 60 wt% Ti_3_C_2_T_x_ | 99.96 | 60 wt% Ti_3_C_2_T_x_ | 99.9996 |
| 80 wt% Ti_3_C_2_T_x_ | 99.997 | 80 wt% Ti_3_C_2_T_x_ | 99.99999 |

Table S2: Comparison of EMI SE *vs.* thickness for different polymer-matrix EMI shielding composites at X-band.

| **Samples** | **Thickness (mm)** | **EMI SE (dB)** | **Ref.** |
| --- | --- | --- | --- |
| MWCNT/ABS | 1.1 | 50 | 1[1] |
| CB/ABS | 1.1 | 20 | 1 |
| MXene/PVA | 0.027 | 44.4 | 2[2] |
| rGO/PI | 0.8 | 21 | 3[3] |
| SWCNT/PS | 1.2 | 18.5 | 4[4] |
| GO/PEDOT:PSS | 1.5 | 69.1 | 5[5] |
| rGO/Fe_2_O_3_/PVA | 0.36 | 20.3 | 6[6] |
| CNT/PPS | 1.5 | 43 | 7[7] |
| Ag NW/cellulose | 0.16 | 48.6 | 8[8] |
| Cu/natural fiber | 0.8 | 24.6 | 9[9] |
| NiCo/PAN-PU | 0.18 | 68 | 10[10] |
| Ti_3_C_2_T_x_/CNFs | 0.047 | 24 | 11[11] |
| d-Ti_3_C_2_T_x_/ANF | 0.012 | 34.71 | 12[12] |
| MXene/CNF | 0.035 | 39.6 | 13[13] |
| MXene/PEDOT:PSS | 0.011 | 42.1 | 14[14] |
| ***R-Ti***_3_***C***_2_***T***_x_***/(ANF/PVA)*** | ***0.12*** | ***46*** | ***This work*** |
| ***D-Ti***_3_***C***_2_***T***_x_***/(ANF/PVA)*** | ***0.12*** | ***70*** | ***This work*** |

*MWCNT: multi-walled carbon nanotubes; ABS: acrylonitrile butadiene styrene; CB: carbon black; PI: polyimide; rGO: reduced graphene oxide; SWCNT: single wall carbon nanotube; PS: polystyrene; PEDOT:PSS: poly(3, 4-ethylenedioxythiophene)-poly(styrenesulfonate); CNT: carbon nanotubes; Ag NW: silver nanowire; PAN: polyacrylonitrile; PU: polyurethane; CNFs: cellulose nanofibers; PPS: poly(phenylene sulfide).

Table S3: Comparison of SSE/t *vs.* density for different polymer-matrix EMI shielding composites at X-band.

| **Samples** | **Thickness (mm)** | **Density**  **(g·cm^-3^)** | **EMI SE (dB)** | **SSE/t**  **(dB·cm^2^·g^-1^)** | **Ref.** |
| --- | --- | --- | --- | --- | --- |
| MWCNT/ABS | 1.1 | 1.05 | 50 | 432.7 | [1] |
| rGO/PI | 0.8 | 0.022 | 21 | 11712 | [3] |
| Ti_3_C_2_T_x_/CNFs | 0.047 | 2 | 24 | 2647 | [11] |
| Ti_3_C_2_T_x_/PEDOT:PSS | 0.012 | 1.89 | 28.2 | 12465 | [14] |
| Graphene/PEI | 2.3 | 0.03 | 22 | 3190 | [15] |
| CNT/cellulose | 2.5 | 0.0323 | 50.8 | 6287 | [16] |
| CF/PAM/wood | 6 | 0.65 | 41 | 105.2 | [17] |
| MWCNT/PLLA | 2.5 | 0.3 | 23 | 308 | [18] |
| rGO-Fe_3_O_4_/PEI | 2.5 | 0.409 | 18 | 176 | [19] |
| Ni fiber/PES | 2.85 | 1.87 | 58 | 108.7 | [20] |
| Graphene/PDMS | 3 | 0.06 | 36 | 2000 | [21] |
| Ti_3_C_2_T_x_/SA | 0.014 | 2.5 | 43.9 | 14830 | [22] |
| Ti_3_C_2_T_x_/CA | 0.026 | 1.35 | 54.3 | 17586 | [22] |
| Ti_3_C_2_T_x_/rGO/epoxy | 2 | 0.03 | 56.4 | 9400 | [23] |
| ***R-Ti_3_C_2_T_x_/(ANF/PVA)*** | ***0.12*** | ***0.423*** | ***46*** | ***9062*** | ***This work*** |
| ***D-Ti_3_C_2_T_x_/(ANF/PVA)*** | ***0.12*** | ***0.423*** | ***70*** | ***13790*** | ***This work*** |

*PEI: poly(ethylene imine); CF: carbon fiber; PAM: polyacrylamide; PLLA: L-polylactic acid; PES: polyethersulfone; PDMS: polydimethylsiloxane; SA: sodium alginate; CA: calcium alginate.

**References**

[1] M. H. Al-Saleh, W. H. Saadeh and U. Sundararaj, “EMI shielding effectiveness of carbon based nanostructured polymeric materials: A comparative study,” *Carbon*, vol. 60, pp. 146-156, 2013.

[2] X. Jin, J. Wang, L. Dai et al., “Flame-retardant poly(vinyl alcohol)/MXene multilayered films with outstanding electromagnetic interference shielding and thermal conductive performances,” *Chemical Engineering Journal*, vol. 380, article 122475, 2020.

[3] Y. Li, X. Pei, B. Shen et al., “Polyimide/graphene composite foam sheets with ultrahigh thermostability for electromagnetic interference shielding,” *RSC Advances*, vol. 5, pp. 24342-24351, 2015.

[4] Y. Yang and M. C. Gupta, “Novel Carbon Nanotube-Polystyrene Foam Composites for Electromagnetic Interference Shielding,” *Nano Letters*, vol. 5, pp. 2131-2134,

[5] Y. Wu, Z. Wang, X. Liu et al., “Ultralight Graphene Foam/Conductive Polymer Composites for Exceptional Electromagnetic Interference Shielding,” *ACS Applied Materials & Interfaces*, vol. 9, pp. 9059-9069, 2017.

[6] B. Yuan, C. Bao, X. Qian et al., “Design of artificial nacre-like hybrid films as shielding to mitigate electromagnetic pollution,” *Carbon*, vol. 75, pp. 178-189, 2014.

[7] X.-P. Zhang, L.-C. Jia, G. Zhang et al., “A highly efficient and heat-resistant electromagnetic interference shielding carbon nanotube/poly(phenylene sulfide) composite via sinter molding,” *Journal of Materials Chemistry C*, vol. 6, pp. 10760-10766, 2018.

[8] T.-W. Lee, S.-E. Lee and Y. G. Jeong, “Highly Effective Electromagnetic Interference Shielding Materials based on Silver Nanowire/Cellulose Papers,” *ACS Applied Materials & Interfaces*, vol. 8, pp. 13123-13132, 2016.

[9] Q. Wang, S. Xiao, S. Q. Shi et al., “Self-bonded natural fiber product with high hydrophobic and EMI shielding performance via magnetron sputtering Cu film,” *Applied Surface Science*, vol. 475, pp. 947-952, 2019.

[10] N. Zhang, R. Zhao, D. He et al., “Lightweight and flexible Ni-Co alloy nanoparticle-coated electrospun polymer nanofiber hybrid membranes for high-performance electromagnetic interference shielding,” *Journal of Alloys and Compounds*, vol. 784, pp. 244-255, 2019.

[11] W. T. Cao, F. F. Chen, Y. J. Zhu et al., “Binary Strengthening and Toughening of MXene/Cellulose Nanofiber Composite Paper with Nacre-Inspired Structure and Superior Electromagnetic Interference Shielding Properties,” *ACS Nano*, vol. 12, pp. 4583-4593, 2018.

[12] H. Wei, M. Wang, W. Zheng et al., “2D Ti_3_C_2_T_x_ MXene/aramid nanofibers composite films prepared via a simple filtration method with excellent mechanical and electromagnetic interference shielding properties,” *Ceramics International*, vol. 46, pp. 6199-6204, 2020.

[13] B. Zhou, Z. Zhang, Y. Li et al., “Flexible, Robust, and Multifunctional Electromagnetic Interference Shielding Film with Alternating Cellulose Nanofiber and MXene Layers,” *ACS Applied Materials & Interfaces*, vol. 12, pp. 4895-4905, 2020.

[14] R. Liu, M. Miao, Y. Li et al., “Ultrathin Biomimetic Polymeric Ti_3_C_2_T _x_ MXene Composite Films for Electromagnetic Interference Shielding,” *ACS Applied Materials & Interfaces*, vol. 10, pp. 44787-44795, 2018.

[15] J. Ling, W. Zhai, W. Feng et al., “Facile preparation of lightweight microcellular polyetherimide/graphene composite foams for electromagnetic interference shielding,” *ACS Applied Materials & Interfaces*, vol. 5, pp. 2677-84, 2013.

[16] L. Q. Zhang, S. G. Yang, L. Li et al., “Ultralight Cellulose Porous Composites with Manipulated Porous Structure and Carbon Nanotube Distribution for Promising Electromagnetic Interference Shielding,” *ACS Applied Materials & Interfaces*, vol. 10, pp. 40156-40167, 2018.

[17] B. Dang, Y. Chen, N. Yang et al., “Effect of carbon fiber addition on the electromagnetic shielding properties of carbon fiber/polyacrylamide/wood based fiberboards,” *Nanotechnology*, vol. 29, article 195605, 2018.

[18] T. Kuang, L. Chang, F. Chen et al., “Facile preparation of lightweight high-strength biodegradable polymer/multi-walled carbon nanotubes nanocomposite foams for electromagnetic interference shielding,” *Carbon*, vol. 105, pp. 305-313, 2016.

[19] B. Shen, W. Zhai, M. Tao et al., “Lightweight, multifunctional polyetherimide/graphene@Fe_3_O_4_ composite foams for shielding of electromagnetic pollution,” *ACS Applied Materials & Interfaces*, vol. 5, pp. 11383-11391, 2013.

[20] X. P. Shui and D. D. L. Chung, “Nickel Filament Polymer-Matrix Composites with Low Surface Impedance and High Electromagnetic Interference Shielding Effectiveness,” *Journal of Electronic Materials*, vol. 26, pp. 928-934, 1997.

[21] Z. Chen, C. Xu, C. Ma et al., “Lightweight and flexible graphene foam composites for high-performance electromagnetic interference shielding,” *Advanced Materials*, vol. 25, pp. 1296-300, 2013.

[22] Z. Zhou, J. Liu, X. Zhang et al., “Ultrathin MXene/Calcium Alginate Aerogel Film for High‐Performance Electromagnetic Interference Shielding,” *Advanced Materials Interfaces*, vol. 6, article 1802040, 2019.

[23] S. Zhao, H. B. Zhang, J. Q. Luo et al., “Highly Electrically Conductive Three-Dimensional Ti_3_C_2_T_x_ MXene/Reduced Graphene Oxide Hybrid Aerogels with Excellent Electromagnetic Interference Shielding Performances,” *ACS Nano*, vol. 12, pp. 11193-11202, 2018.
